# Prevalence and severity of depression in chronic viral hepatitis in Iran

**DOI:** 10.1093/gastro/gou091

**Published:** 2015-02-05

**Authors:** Mojgan Mirabdolhagh Hazaveh, Taraneh Dormohammadi Toosi, Mohsen Nasiri Toosi, Amir Tavakoli, Fatemeh Shahbazi

**Affiliations:** ^1^Rheumatology Research Centre, Vali-asr Hospital, Imam Khomeini Hospital Complex, Tehran University of Medical Sciences, Tehran, Iran; ^2^Gastrointestinal Research Department, Imam Khomeini Hospital, Tehran University of Medical Sciences, Tehran, Iran; ^3^Department of Biology, Payame Noor University, Iran

**Keywords:** chronic hepatitis, hepatitis C, hepatitis B, depression

## Abstract

**Aim:** The aim of this study was to compare the prevalence and severity of depression in chronic hepatitis B (CHB) patients, chronic hepatitis C (CHC) patients, and healthy participants.

**Methods:** Two hundred and fifty-three persons participated in this cross-sectional study between 2011 and 2012 in Imam Khomeini Hospital. The prevalence and severity of depression were assessed using the Hamilton Scale.

**Results:** There was significantly higher prevalence of depression in CHC patients (35.9%) than in healthy participants (11.3%) and CHB patients (19.8%) (both *P < *0.01). However, CHB and CHC patients did not differ significantly in their depression prevalence after excluding inactive hepatitis B surface antigen (HBsAg) carriers (29.3% *vs.* 35.9%; *P > *0.05). Inactive HBsAg carriers differed significantly from CHB patients—excluding inactive HBsAg carriers—in depression prevalence (10.0% *vs.* 29.3%; *P < *0.05). No statistically significant difference was found in depression severity between all groups (*P > *0.05).

**Conclusion:** Depression screening in chronic hepatitis B and chronic hepatitis C patients may be beneficial in disease management.

## Introduction

The incidence of depression is increased in patients with major chronic medical disorders, such as conditions involving immune and inflammatory mechanisms [1]. Hepatitis B virus (HBV) and hepatitis C virus (HCV) infection are the major causes of chronic hepatitis [2, 3]. Depression is a very common complaint among chronic hepatitis patients who do not receive any antiviral therapy [4]. Since depression worsens the outcomes of comorbid physical conditions, an accurate estimation of depression prevalence in chronic hepatitis B and C (CHB, CHC) patients was reported to be needed, in order to decide on the need for depression screening in patients with hepatitis [5–7].

Previous studies showed higher rates of depression in CHC patients [4, 8–13] and CHB patients who did not receive prior anti-viral therapy [14, 15]. Other studies comparing depression prevalence in CHC patients, CHB patients and healthy participants reported differing results [16–18]. This study was performed to further compare the prevalence and severity of depression in CHB patients, CHC patients, and healthy participants.

## Materials and Methods

### Study population and setting

The study protocol was approved by the Institutional Review Board at Tehran University of Medical Sciences. This cross-sectional study was conducted between November 2011 and December 2012. The setting for participants’ recruitment was the hepatitis clinic of Imam Khomeini Hospital, which is a tertiary care medical centre in Tehran. Convenience sampling strategy was used. The investigators explained the design and purpose of the study to participants who met eligibility criteria before obtaining informed consent.

The inclusion criterion for patients was HBV or HCV infection diagnosed at least 6 months previously, using polymerase chain reaction (PCR). Patients with clinical symptoms or signs of cirrhosis or portal hypertension were excluded. Further exclusion criteria were antiviral therapy, any chronic physical or psychiatric disease or malignancy, past history of chronic physical or psychiatric diseases or malignancy, and co-infection with human immunodeficiency virus (HIV), HCV, or HBV. Finally, inactive hepatitis B surface antigen (HBsAg) carriers were defined as CHB patients without liver injuries, identified with liver enzyme tests or liver biopsy.

Healthy participants were hospital staff members free from known physical or psychiatric diseases, and who were not using any drugs or medication.

### Evaluation of depression

The outcomes were the rate and severity of depression for each group. All participants were interviewed by one trained psychologist using a 17-item Hamilton Depression Rating Scale (HAMD-17). A minimum of 20 minutes was allocated to each clinical interview. HAMD-17 includes 17 items (depression mood, feeling of guilt, suicide, insomnia early, insomnia middle, insomnia late, work and activities, retardation psychomotor, agitation, anxiety psychological, anxiety somatic, anxiety psychological, somatic symptoms general, genitalial symptoms, hypochondriasis, loss of weight, insight), aiming to identify the presence and severity of depression. A validity and reliability study was performed and the cut-off score was accepted as 10. Categoric scores for depression severity were determined as: no depression, score <10; mild depression, score 10–13; moderate depression, score 14–17; severe depression, score >17).

Demographic and clinical characteristics of all participants were recorded using a checklist. In addition, CHC patients' clinical characteristics, including serum level alanine aminotransferase enzyme (ALT), viral load, fibrosis stage of liver, genotype of virus, and past history of intravenous (IV) drug abuse were recorded. Viral load and genotype of virus, measured with PCR test, and fibrosis stage of liver were identified with liver biopsy. If there was a history of IV drug abuse, participants were considered to be drug-free if they had not injected in the previous 4 months and recorded with a data collection form.

### Statistical analysis

In this study, the three groups were compared for proportion and severity of depression, using the Pearson χ^2^ test and Kruskal-Wallis test, respectively. Inactive HBsAg carriers were also compared with CHB patients, CHC patients, and healthy participants using the Pearson χ^2^ tests. Either the Pearson χ^2^ test or Fisher’s exact test were used to compare depression prevalence in different subgroups of CHC patients. All *P*-values were two-tailed and a *P < *0.05 was considered significant. SPSS 16.0 was used for statistical analyses.

## Results

Eighty-one CHB patients, 92 CHC patients, and 80 healthy participants met the eligibility criteria and were analysed. Participants’ clinical and demographic characteristics are summarized by group in Table 1.

**Table 1.** Demographic characteristics of patients

Variable	Chronic hepatitis B patients (*n = *81)	Chronic hepatitis C patients (*n = *92)	Healthy participants (*n = *80)
Mean age, year	33.8 ± 12.7	37.5 ± 9.7	35.2 ± 12.3
Female,* n* (%)	19 (23.5%)	12 (12.9%)	15 (18.75%)
Marital status, single,* n* (%)	29 (35.8%)	29 (31.5%)	37 (46.2%)
Educational status,* n* (%)			
Illiterate	16 (19.8%)	2 (2.2%)	2 (2.5%)
Compulsory education	59 (72.8%)	85 (92.4%)	68 (85.0%)
University education	6 (7.4%)	5 (5.4%)	10 (12.5%)
Smoking,* n* (%)	16 (19.8%)	68 (73.9%)	14 (17.5%)
Alcohol consumption,* n* (%)	8 (9.9%)	37 (40.2%)	7 (8.75%)

Nine (11.3%) healthy participants and 49 (28.3%) patients with hepatitis, including 16 (19.8%) CHB patients and 33 (35.9%) CHC patients were depressed. There was a significantly higher rate of depression in patients with hepatitis than in healthy participants (*P = *0.003). In addition, CHC patients showed a higher rate of depression than healthy participants (*P = *0.000) or CHB patients (*P = *0.019). However, CHB patients did not differ significantly from CHC patients in terms of prevalence of depression after excluding inactive HBsAg carriers (29.3% *vs.* 35.9%; *P = *0.457). There was no statistically significant difference between inactive HBsAg carriers and healthy participants (10.0% *vs.* 11.3%; *P = *1.000). Inactive HBsAg carriers and CHB patients—excluding inactive HBsAg carriers—differed significantly in their prevalence of depression (10.0% *vs.* 29.3%; *P = *0.029).

Table 2 shows the proportion of patients with mild, moderate, and severe depression. Comparing CHB patients, CHC patients, and healthy participants, there was no statistically significant difference in the severity of their depression (*P = *0.120).

**Table 2.** Proportion of patients with depression by severity category

Participants	No depression *n* (%)	Mild *n* (%)	Moderate *n* (%)	Severe *n* (%)
Chronic hepatitis B patients (*n = *81)	65 (80.2%)	9 (11.1%)	5 (6.2%)	2 (2.5%)
Inactive HBsAg carriers (*n = *40)	36 (90.0%)	3 (7.5%)	0 (0%)	1 (2.5%)
Chronic hepatitis B patients excluding inactive HBsAg carriers (*n = *41)	29 (70.7%)	6 (14.6%)	5 (12.1%)	1 (2.4%)
Chronic hepatitis C patients (*n = *92)	59 (64.1%)	11 (12%)	14 (15.2%)	8 (8.7%)
Healthy participants (*n = *80)	71 (88.75%)	6 (7.5%)	2 (2.5%)	1 (1.25%)

Among CHC patients, there was a significantly higher rate of depression in smokers (*P = *0.006) and alcohol drinkers (*P = *0.012), but this prevalence was not associated with viral load, HCV genotype, fibrosis stage, or serum ALT level (all *P > *0.05) (Table 3).

**Table 3.** Prevalence of depression in chronic hepatitis C patients

Variable	Category	*n*	Depressed,* n* (%)	*P*-value
Sex	Male	80	28 (35.0%)	0.750
	Female	12	5 (41.7%)	
Marital status	Single	29	12 (41.4%)	0.455
	Married	63	21 (33.3%)	
Smoker	Yes	68	39 (57.3%)	0.006
	No	24	6 (25.0%)	
Alcohol drinker	Yes	37	24 (64.9%)	0.012
	No	55	21 (38.2%)	
IV drug abuser	Yes	52	22 (42.3%)	0.142
	No	40	11 (27.5%)	
Viral load	>10^6^	50	16 (32.0%)	0.356
	≤10^6^	20	8 (40.0%)	
Fibrosis stage	>3	11	6 (54.5%)	0.459
	3	22	8 (36.4%)	
ALT twice the upper limit of normal	Yes	58	19 (32.7%)	0.628
	No	26	10 (38.4%)	
HCV-genotype	Type 1	33	8 (24.2%)	0.067
	Type 3	50	22 (44.0%)	

• ALT = alanine aminotransferase.

## Discussion

Previous studies comparing the prevalence of depression in CHC, CHB patients and healthy participants reported varying results. Carta *et al.* demonstrated that a higher prevalence of major depressive disorder (MDD) could be observed among CHC patients than in CHB patients or controls and the risk of MDD was not statistically different between the CHB group and controls (17.1% *vs.* 13.8%; *P = *0.58) [16]. Qureshi *et al.* showed that both CHC and CHB groups had a higher frequency of some degree of depression (72.6% and 58.6%) than healthy subjects (37.8%), and CHC patients had more depressive features than CHB [17]. However, Ozkan *et al.* reported no significant difference in the rate of psychiatric diagnosis (mainly depression) between the CHB and CHC patient groups [18]. This study indicates that the rate of depression across the combined CHB and CHC patient group including is higher than that among healthy participants (28.3% *vs.* 11.3%), and that CHC patients are more vulnerable to depression than those with CHB (35.9% *vs.* 19.8%). Depressed CHC patients, CHB patients, and healthy participants tend towards lower depression severity scores, but the differences did not reach statistical significance.

Atesci *et al.* found that inactive adult HBsAg carriers exhibited significantly greater depression than healthy controls [19]; however, Arslan *et al.* demonstrated that, in respect of the extent of depression, children with HBV infection were no different from those who were HBsAg carriers, nor from healthy controls [20]. In the present study, after excluding inactive HBsAg carriers, the prevalence of depression increases significantly among CHB patients. Inactive HBsAg carriers are similar to healthy participants in their prevalence of depression (10.0% *vs.* 11.3%).

In addition, the present study analysed the risk factors associated with depression among CHC patients. Smoking and alcohol consumption have increased the rate of depression in CHC patients, while viral load, liver fibrosis stage, HCV genotype, serum ALT level, past history of IV drug abuse, marital status, and gender have not had any influence. Hence infection with HBV or HCV may not be the only cause of higher rates of depression in these patients. The chronic nature of the disease, stigmatization, or patients' perceptions of adverse outcomes of disease may be the additional causes. Depression screening in CHB and CHC patients, especially smokers or alcohol consumers, may be beneficial in disease management.

This study has some limitations, including non-probability sampling and the absence of group matching. It still has some strength: during this study a single interviewer—who was a trained psychologist instead of a trained general practitioner—completed all of the questionnaires; in addition, the questionnaire measures the severity of depression, as well as assessing its presence.

In conclusion, our findings suggest that depression screening in chronic hepatitis B and chronic hepatitis C patients may be beneficial in disease management. However, it is necessary to conduct better-developed studies with adequate sample size to show the effects of depression screening on chronic hepatitis management in this population.

*Conflict of interest statement:* none declared.
